# Data for comparative proteomics of ovaries from five non-model, crustacean amphipods^☆^

**DOI:** 10.1016/j.dib.2015.07.037

**Published:** 2015-08-12

**Authors:** Judith Trapp, Christine Almunia, Jean-Charles Gaillard, Olivier Pible, Arnaud Chaumot, Olivier Geffard, Jean Armengaud

**Affiliations:** a Irstea, Unité de Recherche MALY, Laboratoire d׳écotoxicologie, CS70077, F-69626 Villeurbanne, France; b CEA-Marcoule, DSV/IBITEC-S/SPI/Li2D, Laboratory “Innovative Technologies for Detection and Diagnostic”, BP 17171, F-30200 Bagnols-sur-Cèze, France

## Abstract

Ovaries were taken from five sexually mature amphipods: *Gammarus fossarum, Gammarus pulex, Gammarus roeseli, Hyallela azteca and Parhyale hawaiensis*. The soluble proteome extracted from individual pair of ovaries from five biological replicates was trypsin digested and the resulting peptides were analyzed by high resolution tandem mass spectrometry. The spectra were assigned with four protein sequence databases with different specificities: a RNAseq-derived *G. fossarum* database; a RNAseq-derived *P. hawaiensis* database; both originating from ovaries transcriptome; the *Daphnia pulex* database derived from whole-genome sequencing and the NCBInr database. The best interpretation was obtained for most animals with the specific RNA-seq protein database previously established by means of RNAseq carried out on *G. fossarum*. Proteins identified in the five amphipod species allow defining the core-proteome of female reproductive tissues of the *Senticaudata* suborder. The data accompanying the manuscript describing the database searches and comparative analysis Trapp et al., 2015 [1] have been deposited to the ProteomeXchange with identifiers PXD002253 (*G. fossarum*), PXD002254 (*G. pulex*), PXD002255 (*G. roeseli*), PXD002256 (*H. Azteca*), and PXD002257 (*P. hawaiensis*).

Specifications Table

**Table:** 

Subject area	*Environmental biology*
More specific subject area	*Amphipod comparative proteomics*
Type of data	*MS data, Tables*
How data was acquired	*Data-dependent acquisition of tandem mass spectra using a LTQ-Orbitrap-XL mass spectrometer (Thermo).,*
Data format	*.raw files,.mgf peak lists,.mzid identified files from MASCOT (Matrix science),.xls output data after validation with IRMA software.*
Experimental factors	For each female, the ovary pair was dissected under stereomicroscope magnification, immediately frozen in liquid nitrogen and stored at −80 °C until needed. Proteins were extracted and analyzed by shotgun proteomics.
Experimental features	*The 25 proteomes were briefly run on SDS-PAGE, followed by trypsin proteolysis. Tryptic peptides were analyzed by nanoLC-MS/MS and spectra were assigned with four protein sequence databases.*
Data source location	CEA-Marcoule, DSV-Li2D, Laboratory “Innovative technologies for Detection and Diagnostics”, BP 17171, F-30200 Bagnols-sur-Cèze, France
Data accessibility	**Deposited to the ProteomeXchange with identifiers PXD002253 for*****G. fossarum*****, PXD002309 for*****G. pulex*****, PXD002311 for*****G. roeseli*****, PXD002308 for*****H. Azteca*****, and PXD002310 for*****P. hawaiensis*****(****http://proteomecentral.proteomexchange.org****).**

## 1 **Value of the data**

• The data are a precious resource about an ovary proteome map comparison of five different amphipods from the *Senticaudata* suborder for researchers working on emerging model organisms in the field of ecotoxicology or evolutionary ecology.
• We proposed a new strategy for protein quantification for comparing three species of the *Gammarida* infraorder and two of the *Talitrida* infraorder taking advantage of a restricted database including proteins previously identified after the search with four databases.
• The data have been used to define the core-proteome of five amphipods and elaborate on the most conserved proteins. As described in detail in the accompanying manuscript [1], an overall view of ovary proteome map of female sexually mature of five amphipods is presented.

## 2 Experimental design and data

**Fig. 1:**
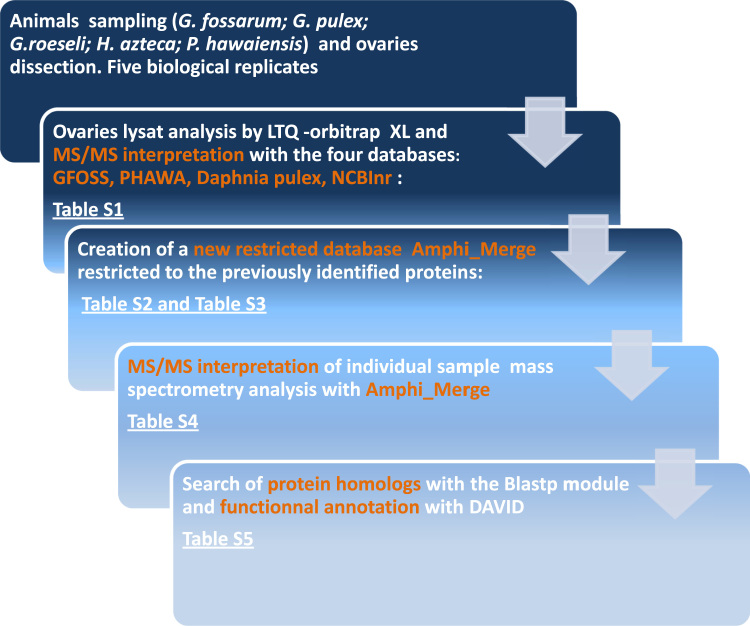
Flowchart of experiments, data processing and refined outputs.

Fig. 1 shows the schematic flowchart of experiments, data processing and results that were presented in.xls tables. s Amphipods were sampled from rivers in mid-eastern France or from laboratory husbandries. Ovaries were taken and then treated for shotgun mass spectrometry analysis. Five biological replicates per species were analyzed, resulting in 25 proteome samples. The peptides from each sample were analyzed by tandem mass spectrometry with an LTQ-Orbitrap-XL spectrometer (Thermo). A first round of MS/MS spectra search was done with four different databases to assign them to tryptic peptide sequences. Two databases derived from RNASeq were used, GFOSS, described by Trapp et al. [2] which is *G. fossarum* specific and PHAWA, *P. hawaiensis* specific [3]. These two databases contain the six frame translation of the sequenced transcriptome. As a consequence, these databases comprised both the true protein sequences and a lot of false translated protein sequences as usually handled by proteogenomics [4,5]. To complete the search, two more databases, the *Daphnia pulex* whole-genome protein sequence database and the non-redundantd database NCBInr were used. In this case, the MS/MS spectra files acquired on the five biological replicates of the same species were merged before spectra assignation. The list of the overall assigned spectra and the peptide characteristics are described in Table S1, while the proteins identified are listed in Table S2. Table S3 summarizes the ratio of each database contribution in terms of spectra assignation. The 2192 identified proteins were then selected to create a specific ovary amphipod restricted database, which was named AMPHI-MERGE. For the second step, spectra assignation of ovary proteome was performed with the AMPHI-MERGE database, for each of the 25 animal proteomes separately. The list of assigned spectra and the corresponding peptide characteristics are described in Table S4 whereas the proteins identified and their spectral count quantitation are listed in Table S5. Then, protein homologs were searched for the resulting identified proteins using the Blastp alignment tool. Homologous proteins were found for almost the entire protein list. Based on their most-closely homologs (same protein GeneID), the detected proteins were grouped together under one protein group. Finally, homolog proteins GeneID were used to associate a function to the detected proteins with the Gene Ontology annotation system. These data were used to define the core ovary proteome of the five amphipods [1].

## 3 Materials and methods

### 3.1 Sampling of animals

The amphipods from the Gammarida infraorder were sampled from rivers in mid-eastern France. They were collected by kick sampling, as previously described [2]. The organisms were determined by phenotypic criteria [6]. *G. pulex* organisms were collected in the Tanche River (latitude, 47°052’815”; longitude: 5°639’305”) while *G. fossarum* and *G. roeseli* organisms were collected in the Bourbre River (45°569’442”; 5°459’115” and 45°716’018”, 5°159’666”, respectively). The organisms from the *Talitrida* infraorder were sampled from laboratory husbandries. Organisms were kindly provided by Bernard Clément for *H. azteca* [7] and Michalis Averof for *P. hawaiensis* [8]. Sexually mature organisms in amplexus were selected. Based on description of the female reproductive cycle [9], only ovaries from females at the end of their reproductive cycle were retrieved. For each female, the ovary pair was dissected under stereomicroscope magnification, immediately frozen in liquid nitrogen and stored at −80 °C until needed. For each species, five biological replicates were performed.

### 3.2 Preparation of biological samples

For protein extraction, ovaries were dissolved in 40 µL LDS sample buffer (Invitrogen), sonicated for 1 min in a transonic 780 H sonicator and boiled for 5 min at 95 °C, essentially as previously described by Trapp et al. [2]. Protein extracts (35 µL) were resolved by SDS-PAGE with a short migration of 10 min at 150 V on 4–12% gradient 10-well NuPAGE (Invitrogen) gels run with MES buffer (Invitrogen) and stained with Coomassie Blue Safe stain (Invitrogen). The whole protein content from each well was extracted as a sole polyacrylamide band. The samples were destained, treated with iodoacetamide, and proteolyzed with Sequencing Grade Trypsin (Roche) using 0.01% ProteaseMAX surfactant (Promega) as described in [10]. The resulting peptide mixtures were diluted 1:20 in 0.1% trifluoroacetic acid. For protein content standardization across species, and based on gel densitometry analysis and pre-testing in nanoLC-MS/MS with a total ion counting procedure, samples were further diluted in 0.1% trifluoroacetic acid for *Gammarus* organisms: 1:20 for *G. fossarum*, 1:15 for *G. pulex* and *G. roeseli*. NanoLC-MS/MS experiments were performed with a LTQ-Orbitrap XL hybrid mass spectrometer (ThermoFisher) coupled to an UltiMate 3000 LC system (Dionex-LC Packings) [11]. On a reverse-phase pre-column C18 PepMap 100 column (LC Packings), 10 µL peptide samples were loaded and desalted online. Peptides were then resolved on a nanoscale C18 PepMapTM 100-capillary column (LC Packings) at a flow rate of 0.3 µL/min with a gradient of CH_3_CN, 0.1% formic acid prior to injection into the ion trap mass spectrometer. Peptides were separated using a 90-min gradient from 5 to 60% solvent B (0.1% HCOOH, 80% CH_3_CN). Solvent A was 0.1% HCOOH, 100% H_2_O. Full-scan mass spectra were measured from m/z 300 to 1800 with the LTQ-Orbitrap XL mass spectrometer in data-dependent mode using the TOP3 strategy. In brief, a scan cycle was initiated with a full scan of high mass accuracy in the Orbitrap followed by MS/MS scans in the linear ion trap on the three most abundant ions.

### 3.3 Protein sequence databases and MS/MS assignments

For interpretation of MS/MS spectra, four databases were used. The National Center for Biotechnology Information nonredundant database (NCBInr) was downloaded on 2015/02/13. This version comprises 59,642,736 entries totaling 21,322,359,704 amino acids. The *Daphnia pulex* protein database, corresponding to the annotation of the whole genome shotgun sequence data ACJG00000000.1 submitted to Genbank, was downloaded on 2015/02/25. This database comprises 30,611 entries totaling 10,015,651 amino acids. The GFOSS protein database, created from RNA-seq data acquired on *G. fossarum*, was as previously described [2]. This database comprises 1,311,444 entries totaling 289,084,257 amino acids. The Parhyale database (PHAWA), obtained after sequencing of ovaries and embryo transcriptomes [3], was downloaded from the Harvard University resources on 2014/11/05. The PHAWA database comprises 1,905,018 sequence entries totaling 277,367,091 amino acids. A restricted database containing all the proteins detected in a previous round of generalist database searches was created and named Amphi_Merge. It comprises 2192 protein sequences totaling 1,053,147 residues. Molecular ion peak lists were extracted with the Mascot Daemon software (version 2.4.0; Matrix Science) using the extract_msn.exe data import filter (Thermo). Data import filter options were set to 400 (minimum mass), 5000 (maximum mass), 0 (grouping tolerance), 0 (intermediate scans), and 1000 (threshold), as previously described [10]. Peptide assignation with MASCOT was done with the following parameters: full trypsin specificity, maximum of two missed cleavages, mass tolerances of 5 ppm on the parent ion and 0.5 Da on the MS/MS, static modification of carboxyamidomethylated cysteine(+57.0215), and oxidized methionine (+15.9949) as dynamic modification. All peptide matches with a MASCOT peptide score below a *p* Value of 0.05 were filtered. Once MS/MS spectra were assigned, peptide lists were parsed with IRMa Batch (IRMa(64) 1.31.1c_javaSurH), released by Laboratoire BGE/EDyP from CEA [12]. The normalized spectral abundance factor (NSAF) for each protein was calculated as the total spectral count divided by the molecular mass expressed in kDa [13].

### 3.4 *In silico* protein mining and functional annotation

The protein sequences certified by MS/MS were used as queries to find the most similar sequences with the BLASTp module from the NCBI website facilities, as described previously [2]. The NCBI gi number from the first NCBInr homolog (*e*-value threshold below 10) was used to merge amphipod protein groups leaving as main identifier the best MASCOT score hit from the AMPHI_MERGE database. For each of these main identifiers the first BLASTp NCBInr hit giving both an Entrez GeneID in gene2refseq and a GO correspondence in gene2go (ftp://ftp.ncbi.nlm.nih.gov/gene/DATA repository) was retrieved to build a matching GeneID list. Protein groups were then classified into GO categories by means of the Database for Annotation, Visualization and Integration Discovery (DAVID) based on the matching Entrez GeneIDs. GOTERM_CC_1 (CELLULAR COMPONENT), GOTERM_BP_1 (Biological Process) and GOTERM_MF_1 are analyzed at their first level.
